# Negativation of *Trypanosoma cruzi* PCR within Six Months after Treatment of a Child with Nifurtimox

**DOI:** 10.1371/journal.pntd.0003667

**Published:** 2015-05-07

**Authors:** Lauren Pull, Feriel Touafek, Luc Paris, Guillaume Le Loup, Laurent Brutus, Jean-Yves Siriez

**Affiliations:** 1 Service d’Accueil des Urgences Pédiatrique, Hôpital Robert-Debré-Assistance Publique-Hôpitaux de Paris, Paris, France; 2 Laboratoire de Parasitologie et de Mycologie, Hôpital Pitié-Salpêtrière-Assistance Publique-Hôpitaux de Paris, Paris, France; 3 Service des Maladies Infectieuses et Tropicales, Hôpital Tenon-Assistance Publique-Hôpitaux de Paris, Paris, France; 4 UMR216 “Mère et enfant face aux infections tropicales,” Institut de Recherche pour le Développement-Université Paris Descartes, Paris, France; Emory University, UNITED STATES

## Case Description

This case report concerns a male child born in 2002 in Santa Cruz, Bolivia. His parents had been positive for *T*. *cruzi* since 1999 and had never been treated until they moved to France with their children in 2004. The boy has two sisters, both negative for Chagas disease. In March 2009, after a confirmation of the diagnosis of Chagas disease in both parents at Tenon Hospital (Paris), the boy tested positive for anti-*T*.*cruzi* antibodies by indirect immunofluorescence (IIF) (200–400, Table 1) (Slide Immunofluor Chagas, Biocientifica SA, Buenos Aires, Argentina) and enzyme-linked immunosorbent assay (ELISA) (Chagatest Elisa rec v 3.0, Wiener lab, Rosario, Argentina) (index 7.6, Table 1).

**Table 1:** 

	Before treatment start	Day 15 after treatment start	Day 30 after treatment start	Day 45 after treatment start	Day 60 after treatment start = End of treatment	Month 5 after treatment start	Month 8 after treatment start	Month 12 after treatment start	3 years and 5 months after treatment start	5 years and 5 months after treatment start
Dates	25/03/09	17/09/09	30/09/09	14/10/09	04/11/09	12/02/10	27/04/10	03/09/10	13/02/13	10/09/14
Weight (kg)	26.3	nd	25	25	25.1	28.6	28	30.8	44	53
Hemoglobin (g/dL)	13.0	12.6	11.8	12.3	11.7	10.9	11.2	12.5	12.6	nd
Leucocytes	10,200	8,800	8,300	7,000	4,500	6,700	7,800	7,800	6,200	nd
Platelets/mm3	338,000	275,000	222,000	226,000	211,000	283,000	255,000	183,000	226,000	nd
aspartate aminotransferase (ASAT) (IU)	24	22	23	29	nd	24	26	25	21	nd
alanine aminotransferase (ALAT) (IU)	13	18	14	19	nd	22	22	25	23	nd
Total bilirubine (μmol/L)	10	14	15	14	nd	6	10	13	13	nd
Creatinine (μmol/L)	27	36	34	61	29	30	36	38	31	nd
IIF titre: Positive if ≥ 100	200–400	nd	nd	nd	nd	200	nd	400	200	100
ELISA index: Positive if > 1	7.6	nd	nd	nd	nd	7.3	nd	6.9	4.43	3.068
PCR extraction by chelex*	2 positive	nd	nd	nd	1 positive	1 equivocal	negative	negative	negative	negative
	Ct 27/27/nd*				Ct 38/neg/neg	Ct 43/neg/neg					
PCR conclusion	strongly positive	nd	nd	nd	positive†	low positive	negative	negative	negative	negative

nd: not done

* The results n/n/n give the cycle threshold (Ct) where each amplification (made in triplicate) is positive. The PCR is negative if Ct > 45, equivocal if 40 < Ct < 45, and positive if Ct < 40.

^†^: Significant lowering of detected DNA quantity (4 Log10)

Furthermore, real-time PCR in blood was positive for *T*. *cruzi* satellite DNA (Table 1). For this test, DNA was manually extracted from 400 μl of 5 ml of guanidine—ethylenediaminetetraacetic acid (EDTA) blood using CHELEX 100 reagent (Bio-Rad). The target used was a portion (166 pb) of a repeated sequence (GenBank AY520036.1) of *T*. *cruzi* satellite DNA. The real-time PCR was performed in triplicate with a 25-μl reaction volume, including 5μl of DNA extract, on TaqMan 7500 fast real-time PCR systems (Applied Biosystems) using the primers Cruzi 1 (forward primer ASTCGGCTGATCGTTTT CGA 3ʹ) and Cruzi 2 (reverse primer 5ʹAATTCCTCCAGCAGCGGATA 3ʹ) and the probe Cruzi 3 (6-carboxyfluorescein-5ʹCACACACTGGACACCAA 3ʹ-MGB) (minor groove binder) and 1xTaqMan fast master mix without uracil-DNA glycosylase (UNG) (Applied Biosystems) [1]. A noncompetitive internal control of amplification composed of an exogenous DNA (Applied Biosystems TaqMan Exogenous Internal Positive Control Reagents) was included in each PCR reaction to distinguish true target negatives from PCR inhibition, and a nontemplate control was included in each run as a real-time PCR negative control. A sample was considered positive for *T*. *cruzi* when the internal control was efficiently amplified, and the threshold cycle (Ct) for the *T*. *cruzi* target was <45. The Ct for a given sample was the first cycle of the PCR reaction in which fluorescence was detected above the baseline.

The pretreatment clinical examination (weight 26.3 kg; height 123 cm), chest X-ray, 12-lead electrocardiogram (ECG), and echocardiogram were all normal. In particular, there were no cardiac or abdominal signs or symptoms. Treatment began on September 5, 2009, with 12 mg/kg/day of nifurtimox in three divided doses for 60 days. The child tolerated the treatment well, reported no gastrointestinal symptoms, and demonstrated neither excitability nor irritability. Over the first month of treatment, the child lost weight (1.3 kg, 4.9%, Table 1) but regained all of it about a month after treatment completion. A mild cutaneous maculopapular rash with pruritus developed at 6 weeks of treatment but did not result in treatment discontinuation. Biological controls were performed once every 15 days during the treatment period and then at 3, 6, and 10 months after treatment (Table 1). Most biological values stayed constant throughout the treatment, with the exception of a reduction in leukocytes that remained in the normal values for the age. PCR was found to be negative at the 6-month post-treatment assessment (Table 1). We then lost sight of the child for 2 years. When we were finally able to examine him again, 3 years and 5 months after starting the treatment, his clinical examination (weight 44 kg, Table 1) and complementary exams were normal. At that time, PCR remained negative, but serology stayed positive (IIF 200, ELISA 4.43). A new control (Table 1) confirmed negative PCR and decrease of the serologic values (IIF 100, ELISA 3.068, >50% titer reduction compared with pretreatment level 5 years and 5 months after treatment).

## Discussion

In Europe, Chagas disease can be considered as an emerging problem, and several cases have been identified in children. Spain in particular sees significant immigration from Latin America; e.g., 1,800,000 immigrants estimated in 2009. One study in that country found three cases of congenital infection among 1,350 pregnant women of Latin American origin [2], but another found no cases in a cohort of 108 children aged 0 to 14 years and either from Latin America or born in Spain to mothers coming from endemic areas for Chagas disease [3]. In Switzerland, congenital transmission was diagnosed in four newborns and five children between 1 and 11 years of age at the early indeterminate phase of the chronic stage of Chagas disease [4]. In Italy, five children adopted from Bolivia were found to be seropositive for *T*. *cruzi*, as were a number of children among 266 migrants tested for *T*. *cruzi* [5]. Methods of diagnosis and adverse effects of treatment were rarely detailed in these studies. 45 cases of Chagas disease in children have been recently reported from Spain and Switzerland. The diagnostic procedure included microscopic blood examination, in-house real-time polymerase chain reaction, and serological testing. 35 children received benznidazole, five received nifurtimox, and one received both drugs consecutively. At 2 years, five patients presented negative serology, 17 showed a serological titer reduction <50%, and seven had a ≥50% titer reduction [6]. According to recent estimations, there may be as many as 1,500 *T*. *cruzi*-infected individuals living in France. Between 1980 and 2007, there were an estimated 20 to 70 cases of congenital infection annually and potentially 235 adopted children who had Chagas disease [7]. To our knowledge, the case that we report here is the first one diagnosed in France and reported in the literature.

In our patient, specific anti-*T*. *cruzi* antibodies were assessed by two concordant serological methods (IIF and ELISA) [8]. Detection of parasite DNA by PCR was also positive before treatment. The irregular release of parasites into the blood of infected hosts during the chronic phase largely explains why direct and indirect methods used for parasite detection (hemoculture and xenodiagnosis) often show low sensitivity. A study showed that parasitic loads measured by quantitative PCR were correlated to patient age at the time of diagnosis and higher in younger children [9]. Two studies found that detection of parasite DNA by PCR may lay between 80% and 100% in chronically infected children [10,11].

As are most of the children in reported cases, our patient was in the chronic indeterminate phase of Chagas disease; i.e., he had no complaints or signs of cardiac or digestive impairment and no abnormalities on the chest X-ray, the ECG, or the echocardiogram [12]. However, a 10% incidence of ECG abnormalities (typically right bundle branch block with left anterior hemiblock) has been observed among infected children less than 12 years old, indicating that a significant proportion of infected children exhibit early signs of cardiomyopathy [13]. To our knowledge, there are no studies indicating the prevalence of gastrointestinal complications of the disease in children. Central nervous involvement is rather rare in childhood but more frequent and severe in children under 2 years of age in the acute phase of the disease [14].

The purpose of antiparasitic treatment (nifurtimox or benznidazole) is to clear the infection and improve the effectiveness of the immune response. Although the effectiveness of benznidazole in children has been demonstrated [15], there is very little information available on nifurtimox efficacy and on the pharmacokinetics and pharmacodynamics of both drugs in this population [16]. To date, dosing decisions in children have been made on the basis of the scarce data available from the adult population. Neither nifurtimox nor benznidazole are registered in France, and gaining access to them can be difficult; we obtained nifurtimox from WHO headquarters in Geneva for the treatment of the case presented here. During treatment, our patient experienced transitory loss of weight (-1.3 kg) and a mild cutaneous eruption that did not necessitate treatment discontinuation. In a study of 168 congenitally infected children aged 0 to 10 years and treated with nifurtimox (10–15 mg/kg/day for 60 days), the most frequent adverse effects were behavioral changes and excitability (11%), anorexia and weight loss (9%), nausea or vomiting (6%), and finally allergic dermatitis [17]. One of the largest studies of children treated with benznidazole (2,804 children aged 1 to 18 years) found that 18% of the subjects experienced allergic skin reactions, 9% nausea and vomiting, 6% neuromuscular disorders, and 2% serious side effects (toxic epidermal necrolysis and Stevens-Johnson Syndrome) [18]. Serious adverse reactions have been reported in adults treated by nifurtimox [19] but are rarely seen in children. The absence of a pediatric formulation increases the risk of under- or overdosing in children. Therefore, the current development of a dispersible 12.5-mg tablet of benznidazole for the treatment of congenitally infected babies is likely to improve dosing accuracy in children.

In our case, DNA detection by PCR became negative 3 to 6 months after the end of treatment, but anti-*T*. *cruzi* antibodies persisted. The decrease of antibody titers is usually a long process in recent chronic phase cases. A multinational study found that the rapidity of seroconversion varied widely between Central and South American populations. In that study, 87% of treated children in a Central American group (Honduras) had seroconverted at 18 months post-treatment, whereas none or very few of the children in a South American group (Bolivia) had done so in the same time frame [18]. A new perspective on Chagas disease diagnosis was opened by the use of PCR. Two studies, totaling 98 children aged 0 to 15 years and in the recent chronic phase, have evaluated PCR assay for monitoring *T*. *cruzi* infection after chemotherapy with nifurtimox [11,20]. In those studies, there was no negative seroconversion of conventional serology in the 36 months after the end of treatment, but PCR became negative in 87% to 90% of the children. Compared to conventional serological methods, PCR may have higher sensitivity for detecting therapeutic failures and could be employed as a tool for early ascertainment of cure.

Beyond medical shortcomings, the screening, diagnosis, and care of children with Chagas disease in nonendemic countries is challenging, mainly because of the poor awareness and training of physicians in this field and the barriers to healthcare access that migrant populations, often with illegal immigrant status, have to face [21]. Anti-*T*. *cruzi* antibodies should be searched for in the newborns of infected mothers, in immigrant children native to or with parents from Latin America, and in adopted children from these regions. Physicians of nonendemic countries must be more aware of Chagas disease, and reference centers are necessary to support patients. Additionally, comparative trials are urgently needed to determine the efficacy and safety of both nifurtimox and benznidazole in children.

**Key Learning Points**

- In Europe, Chagas disease in children is very uncommon and always imported.
- In European countries with high immigration from Latin America, the incidence of Chagas disease is probably underestimated, partly because of the poor awareness and training of physicians in this field.
- Anti-*T*. *cruzi* antibodies should be systematically searched for in newborns of infected mothers, in immigrant children native to or with parents from Latin America, and in adopted children from these regions.
- Comparative trials are urgently needed to determine the efficacy and safety of both nifurtimox and benznidazole in children.
